# Synthesis of multivalent polymer–aptamer conjugates with enhanced inhibitory potency

**DOI:** 10.2147/IJN.S174673

**Published:** 2018-09-07

**Authors:** Jacob T Martin, Marc Douaisi, Ammar Arsiwala, Manish Arha, Ravi S Kane

**Affiliations:** 1 Koch Institute for Integrative Cancer Research, Massachusetts Institute of Technology, Cambridge, MA, USA; 2 Center for Biotechnology and Interdisciplinary Studies, Rensselaer Polytechnic Institute, Troy, NY, USA; 3 School of Chemical & Biomolecular Engineering, Georgia Institute of Technology, Atlanta, GA, USA, ravi.kane@chbe.gatech.edu

**Keywords:** polyvalency, grafting, oligonucleotide, polyglutamic acid

## Abstract

**Purpose:**

We are interested in designing a modular strategy for creating potent multivalent ligands, which frequently can be used as effective inhibitors of undesired biomolecular interactions. For example, such inhibitors might prevent the self-assembly of bacterial toxins or the attachment of a virus to its host cell receptors.

**Methods:**

We used a biocompatible polyamino acid polymer as a scaffold for grafting multiple copies of an oligonucleotide aptamer (OA). Specifically, the carboxylates on the side chains of polyglutamic acid (PGA) were modified with a thiol-reactive linker, *N*-aminoethyl maleimide (AEM), and thiol-functionalized OAs were attached to the maleimide moieties. The resulting conjugates were tested for their ability to compete with and inhibit the binding of unconjugated monovalent OAs to the target cell receptor.

**Results:**

Multivalent PGA–OA conjugates with low, medium, and high valency were successfully prepared. The varying valency and successful purification to remove unconjugated OAs were confirmed by polyacrylamide gel electrophoresis. The resulting purified conjugates inhibited the binding of unconjugated monovalent OAs, and the measured half maximal inhibitory concentration (IC_50_) values corresponded to a 38–88-fold enhancement of potency on a per-aptamer basis, relative to OA alone.

**Conclusion:**

Multivalent conjugation of OA ligands has potential as a generally useful way to improve the potency of the interaction between the ligand and its target receptor. We have demonstrated this principle with a known OA as a proof of concept as well a synthetic strategy that can be used to synthesize multivalent conjugates of other OAs.

## Introduction

Oligonucleotide aptamers (OAs) are a class of biological macromolecular ligands with features that offer several advantages over peptide and protein ligands,^1^–^3^ especially in the context of developing antagonistic therapeutics. In comparison with the small size and molecular weight of peptides, OAs are typically larger,^4^ which may allow greater contact area between the ligand and the target receptor, thereby enhancing their ability to inhibit other molecular interactions with the target receptor. Increased contact area could also potentially allow greater affinity and specificity, reducing off-target binding. Relative to peptides or proteins, nucleic acids are considered less immunogenic,^3^ which may be an important consideration for seeking Food and Drug Administration (FDA) approval. One example of an OA therapeutic is pegaptanib, a prescription treatment for neovascular age-related macular degeneration. OA synthesis can be scaled up relatively easily with low-cost enzymatic reactions and is amenable to “good manufacturing practice” (GMP) production.^3^ In sum, the desirable molecular properties such as size and affinity, manufacturability, and low immunogenicity are features that make OAs useful for therapeutic design.

Despite the high affinity that an OA ligand might exhibit toward a particular receptor, there are biological situations in which high monovalent affinity may not be enough to achieve the desired effect. For example, many biological interactions are multivalent in nature, involving multiple copies of ligands interacting simultaneously with multiple copies of receptors.^5^ An easily identifiable example is that of a virus particle such as the influenza virus, which is covered with hundreds of hemagglutinin proteins that bind to sialic acid molecules on host cells. The resulting multivalent interaction is characterized by an avidity that can be much stronger than the individual monovalent affinity.^5^ In order to design a therapeutic to compete with such multivalent biological interactions, it may be advantageous to create multivalent ligands.^5^–^7^ For instance, we have previously shown that peptide ligands with very low affinity and no monovalent inhibitory efficacy could nonetheless become potent inhibitors of anthrax toxin assembly when displayed multivalently on polymeric scaffolds.^8^,^9^ While these prior studies explored many of the design principles of multivalent ligand display that enhanced the resulting inhibitor efficacy, they did not explore the use of more potent ligands, such as OAs. Therefore, in the current study, we sought to test for the ability to create multivalent conjugates that displayed multiple copies of OA ligands on a biocompatible polymeric scaffold.

## Methods

### Multivalent conjugate synthesis and purification

#### Activation of polymer scaffolds by reaction with N-aminoethyl maleimide

**Figure 1:**
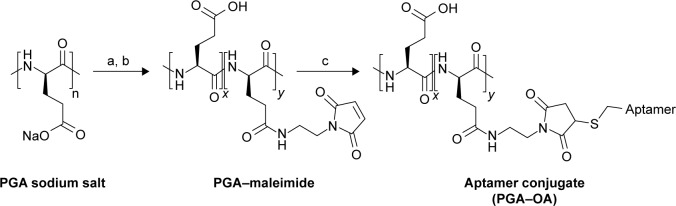
Synthesis scheme for multivalent PGA–OA conjugates. **Notes:** (a) DMTMM and AEM; (b) dialysis; and (c) thiol-OA. The multivalent conjugate is a random copolymer composed of unconjugated monomers (*x* repeat units) and aptamer conjugates (*y* repeat units). **Abbreviations:** AEM, *N*-aminoethyl maleimide; DMTMM, 4-(4,6-dimethoxy-1,3,5-triazin-2-yl)-4-methylmorpholinium tetrafluoroborate; OA, oligonucleotide aptamer; PGA, polyglutamic acid.

The procedure used to activate the biocompatible polymeric PGA scaffolds was based on a modification of previously published protocols for the multivalent attachment of amino-PEG-azide to PGA^10^ and sonic hedgehog protein to hyaluronic acid via thio-ether coupling^11^, and is shown in Figure 1.^11^ PGA with a molecular weight of 120 kDa was purchased from Alamanda Polymers (Huntsville, AL, USA). Using degassed pH 6.5 2-(*N*-morpholino)ethane-sulfonic acid (MES)-buffered saline (MBS) with 10 mM ethylenediaminetetraacetic (EDTA) acid as a buffer, PGA stocks were prepared at 13.2 mg/mL (100 mM glutamate monomer). 4-(4,6-Dimethoxy-1,3,5-triazin-2-yl)–4-methyl-morpholinium tetrafluoroborate (DMTMM; Sigma-Aldrich Co., St Louis, MO, USA) was added at 150 mol% relative to glutamate monomer followed by *N*-aminoethyl maleimide (AEM; Sigma-Aldrich Co.) at 33 mol% relative to glutamate monomer. The reaction was maintained at room temperature for 2 hours. To remove unreacted DMTMM and AEM, the activated polymer scaffolds were then dialyzed extensively against pH 6.5 MBS with 10 mM EDTA, at 4°C to prevent hydrolysis of the maleimide.^12^,^13^ After dialysis, the percent maleimide coupling to the scaffold was estimated by measuring the concentration of AEM in the sample by ultraviolet radiation (UV) absorbance at 290 nm.

#### Activation of aptamer 5′ thiol by the removal of disulfide protecting cap

The single-stranded DNA aptamer “sgc8c”,^14^,^15^ as well as a sequence-scrambled version of the OA (SOA), was purchased from Integrated DNA Technologies, Inc. (Coralville, IA, USA) with a 5′-thiol functionality. The thiol was protected by a disulfide-linked hexane cap. To expose the free thiol, the OA and SOA stocks were incubated with 10 mM tris(2-carboxyethyl)phosphine (TCEP) for 1 hour at pH 6.5 in degassed MBS. The cleaved hexane-thiol cap was then removed from the OA-thiol solution by passing through a 7 K MWCO Zeba™ Spin Desalting Column (Thermo Fisher Scientific, Waltham, MA, USA) equilibrated in Ph 6.5 degassed MBS containing 10 mM EDTA.

#### Conjugation reactions of activated aptamer-thiol with PGA–maleimide

**Figure 2:**
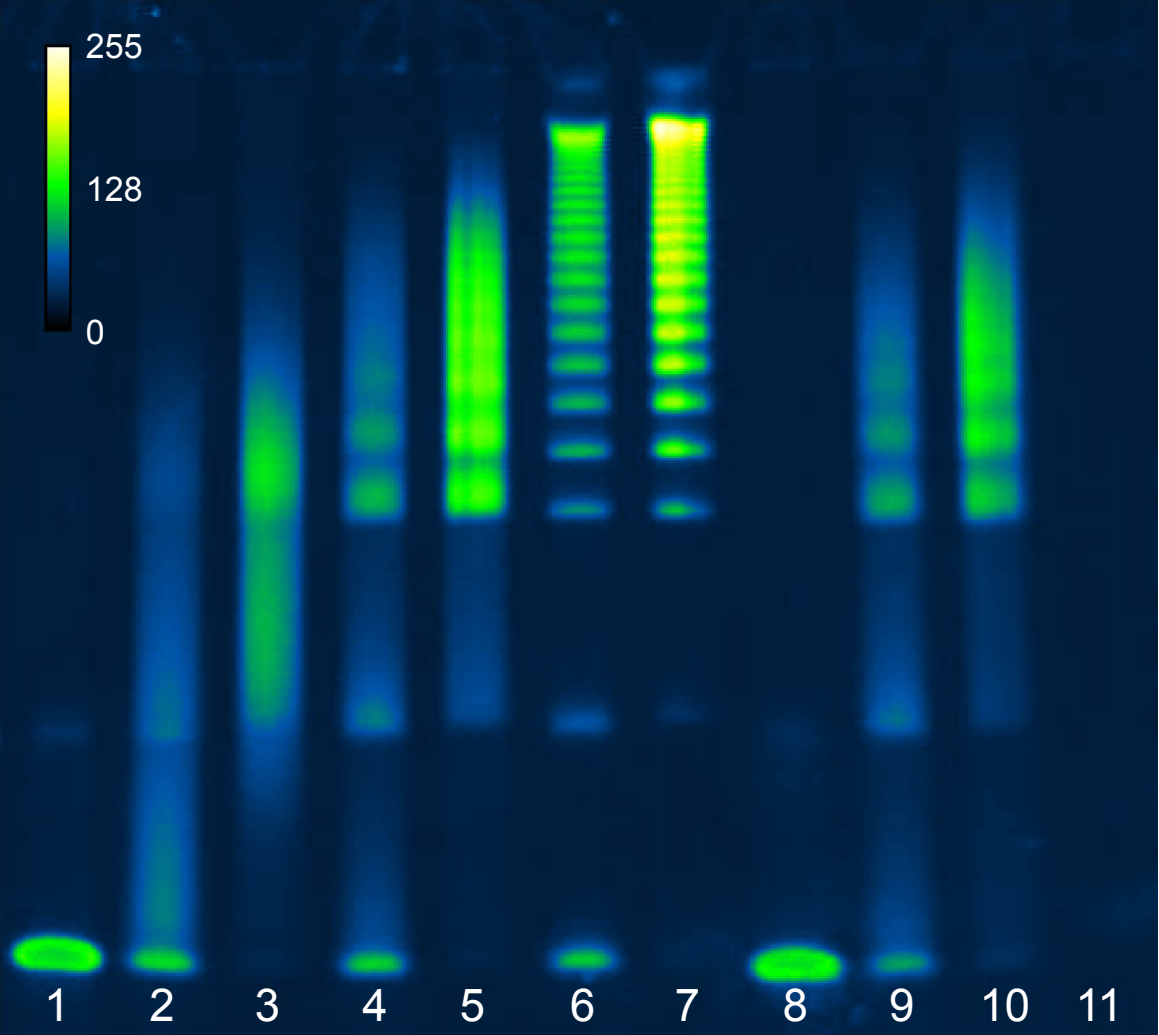
Characterization of PGA–OA conjugation reagents and reaction products before and after purification by gel electrophoresis. **Notes:** SYBR^®^ Gold-stained 4%–20% acrylamide TBE-PAGE of ssDNA aptamer sgc8c conjugation reactions with PGA–maleimide before and after purification by SEC (pseudocolor, scale inset top left). Lanes from left to right: (1) unconjugated OA-thiol; (2 and 3) PGA–OA conjugate (0.5 mol% OA in reaction), before and after SEC purification, respectively; (4 and 5) PGA–OA conjugate (2.5 mol% OA in reaction), before and after SEC purification, respectively; (6 and 7) PGA–OA conjugate (12.5 mol% OA in reaction), before and after SEC purification, respectively; (8) unconjugated SOA-thiol; (9 and 10) PGA–SOA conjugate (2.5 mol% SOA in reaction), before and after SEC purification, respectively; and (11) PGA–maleimide unreacted. **Abbreviations:** OA, oligonucleotide aptamer; PGA, polyglutamic acid; SEC, size-exclusion chromatography; SOA, scrambled OA; TBE-PAGE, tris-borate EDTA polyacrylamide gel electrophoresis.

The deprotected 5′ thiol OAs were then added to the maleimide-modified scaffold at 0.5, 2.5, or 12.5 mol% relative to the glutamate monomers, which roughly corresponds to 1.7, 8.3, or 41.7 mol% relative to the linker maleimides. The reaction buffer was degassed pH 6.5 MBS containing 10 mM EDTA, and the concentration of OAs was maintained as high as possible, typically in the range of 100–200 µM. The reaction was left at ambient temperature for 3 days before checking the conjugation efficiency by polyacrylamide gel electrophoresis (PAGE). Under these conditions, the multivalent products were observed to be the mixtures of multiple species of conjugates with varying degrees of valency as indicated by the multiple bands observed by PAGE (Figure 2).

#### Purification of multivalent products by size-exclusion chromatography (SEC)

Unreacted OA was removed from the multivalent PGA–OA conjugate by SEC on a Superdex 200 prepacked column (GE Life Sciences, Marlborough, MA, USA). The fractions containing PGA–OA, which were free of unconjugated OA, were combined and reconcentrated using 50 K MWCO centrifugal spin filters (EMD Millipore, Billerica, MA, USA).

### Multivalent conjugate characterization

#### PAGE

PAGE was performed using precast 4%–20% TBE gels (Thermo Fisher Scientific). After electrophoresis, gels were stained using SYBR Gold (Thermo Fisher Scientific) and the bands were imaged with a fluorescent gel imager (Bio-Rad Laboratories Inc., Hercules, CA, USA).

#### Binding inhibition assay

Biotinylated sgc8c OA was purchased from Integrated DNA Technologies, Inc. The ability of monovalent nonbiotinylated OA or multivalent PGA–OA to inhibit OA–biotin binding was tested by first incubating serial dilutions of the inhibitors with MOLT-4 cells (T-cell acute lymphoblastic leukemia, ATCC CRL-1582) for 30 minutes, followed by washing and incubation with 100 nM OA–biotin for 30 minutes more. The cells were then washed again and incubated with streptavidin–phycoerythrin (SA–PE) for another 30 minutes, before a final wash and fixation with paraformaldehyde. The SA–PE signal was detected by flow cytometry (LSRII; Becton Dickinson, Franklin Lakes, NJ, USA).

## Results and discussion

We synthesized multivalent conjugates of a known OA, sgc8c,^14^,^15^ on a polyglutamic acid (PGA) scaffold. This OA was discovered by screening ssDNA for binders of T-cell acute lymphoblastic leukemia cells, and the resulting ligand was pared down to a length of 42 bases.^15^–^17^ PGA was chosen as a scaffold because we^8^ and others^18^ have previously used the scaffold for peptide ligand display, and this polymer is expected to be more biocompatible than other polymer scaffolds.^15^,^19^ We used a maleimidethiol coupling strategy to attach 5″-thiol-modified sgc8c to the PGA scaffold, as shown in Figure 1.

PGA–maleimide was mixed with the sgc8c OA or a scrambled sequence of sgc8c (SOA) as a negative control. Freshly TCEP-treated OA-thiol was added at molar ratios 0.5%, 2.5%, or 12.5% OA to glutamate monomer, and SOA-thiol was added at 2.5 mol%. As shown in Figure 2, the reaction products were polydisperse and the resulting conjugate size and valency could be tuned by varying the molar ratio of OA-thiol to polymeric scaffold. We presume that the multiple species visible in each lane with reaction products are indicative of a variation in the resulting conjugation valency. Remaining unreacted OA or SOA was successfully removed by SEC from the multivalent PGA conjugate, as shown in lanes 3, 5, 7, and 10. The same total amount of OA or SOA was loaded in each well, which is why the individual bands appear slightly brighter in the post-SEC lanes. Since various multivalent species from each purified reaction were not easily separated from each other by SEC, the samples were not separated further prior to testing for their inhibition potency.

**Figure 3:**
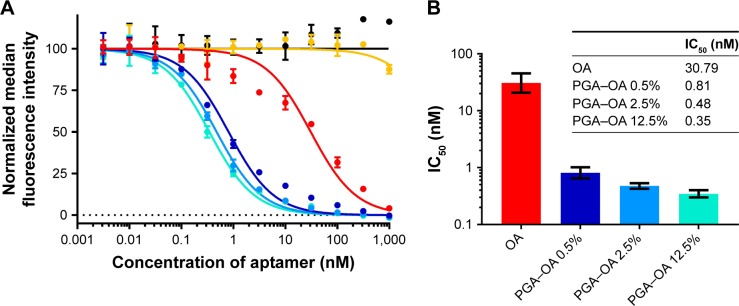
Characterization of the inhibitory potency of monovalent OA and multivalent PGA–OA conjugates. **Notes:** (**A**) Characterization of the inhibition of binding of OA–biotin to MOLT-4 T cells by multivalent PGA–OA conjugates (target 0.5 mol% OA ●, target 2.5 mol% OA ●, and target 12.5 mol% OA ●), multivalent PGA–SOA control (target 2.5 mol% SOA ●), monovalent unconjugated OA (●), or monovalent unconjugated SOA (●). The target mole percentage given for the multivalent samples indicates the molar ratio of OA to glutamic acid repeat units in the reaction mixture. OA–biotin was detected using fluorescent SA–PE conjugates. Curves are nonlinear regression best fits of the data set from each sample, calculated and plotted with GraphPad Prism 7, and error bars indicate the SD of the replicate measurements. Some standard deviations are narrow enough that the corresponding error bars are not displayed by the software. (**B**) The IC_50_ of each sample was determined from the nonlinear regression curves, and the results are listed in the table. The data are also plotted as a bar graph, with the error bars indicating the 95% CI of the nonlinear regression analysis. **Abbreviations:** CI, confidence interval; IC_50_, half maximal inhibitory concentration; OA, oligonucleotide aptamer; PGA, polyglutamic acid; SA–PE, streptavidin–phycoerythrin; SOA, scrambled OA.

Next, we compared the ability of monovalent OA and multivalent PGA–OA to inhibit the labeling of cells with monovalent OA–biotin via a dose–response-binding competition assay. OA–biotin was detected by SA–PE on a flow cytometer, and the median fluorescence intensity (MFI) of the SA–PE signal was normalized to the signal from cells without inhibitor. The dose–response data are plotted in Figure 3A, along with curves fit by nonlinear regression analysis. Half maximal inhibitory concentration values for each sample were calculated from the curves and are plotted in Figure 3B. The data and analysis indicate that on a per-OA basis, all three PGA–OA conjugate samples exhibited a greater inhibitory potency than monovalent OA. The enhancement of inhibitory efficacy ranged from 38- to 88-fold for the conjugates with the lowest to the highest valency PGA–OA. In contrast, sequence-scrambled versions of the OA did not inhibit binding, either monovalently or multivalently.

## Conclusion

We have presented a general method for grafting multiple OAs to linear polymer scaffolds. Due to the advantages of OAs over peptide ligands, we expect that multivalent displays of OAs may prove to be more useful than monovalent OAs or multivalent peptides for inhibiting other biomolecular interactions, especially when those interactions are themselves multivalent. This approach could be useful in a broad range of contexts where potent inhibition is desired, such as for the prevention of viral cell attachment. Such multivalent presentations of OAs may also be useful in a wide variety of additional applications in which strong avidity and specificity are desired, ranging from targeted drug delivery to imaging.^6^,^7^,^20^,^21^
